# (5*R*)-Ethyl 6-benzyl-8,8-dimethyl-7,9-dioxo-1-oxa-2,6-diaza­spiro­[4.4]non-2-ene-3-carboxyl­ate

**DOI:** 10.1107/S1600536809042433

**Published:** 2009-10-28

**Authors:** Yaser Bathich, Mohd Fazli Mohammat, Ahmad Sazali Hamzah, Jia Hao Goh, Hoong-Kun Fun

**Affiliations:** a Institute of Science, Universiti Teknologi MARA, 40450 Shah Alam, Selangor, Malaysia; b X-ray Crystallography Unit, School of Physics, Universiti Sains Malaysia, 11800 USM, Penang, Malaysia

## Abstract

In the title compound, C_18_H_20_N_2_O_5_, the pyrrolidine ring adopts an envelope conformation with the C atom bonded to the methyl groups as the flap. The dihydro­isoxazole ring is essentially planar (r.m.s. deviation = 0.041 Å) and forms a dihedral angle of 65.19 (6)° with the phenyl ring. In the crystal, neighbouring mol­ecules are linked into chains along [110] by inter­molecular C—H⋯O hydrogen bonds and weak C—H⋯π inter­actions involving the phenyl ring.

## Related literature

For general background and applications of the title compound, see: Carmely *et al.* (1990 ▶); Manero *et al.* (2006 ▶); Sauleau & Bourguet-Kondracki (2005 ▶). For a related structure, see: Hamzah *et al.* (2006 ▶). For ring conformations and ring puckering analysis, see: Boeyens (1978 ▶); Cremer & Pople (1975 ▶). For bond-length data, see: Allen *et al.* (1987 ▶). For the stability of the temperature controller used for the data collection, see: Cosier & Glazer (1986 ▶).
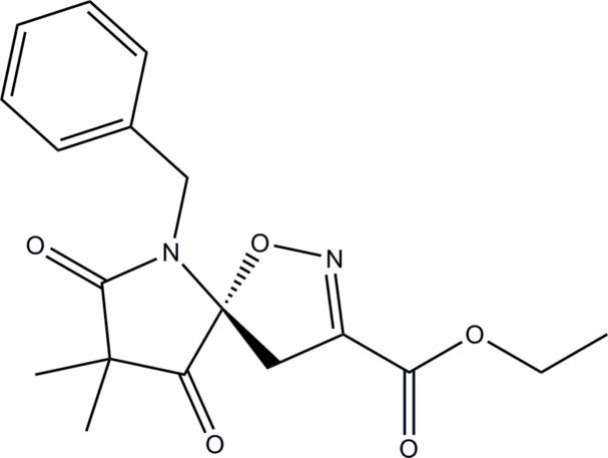


## Experimental

### 

#### Crystal data

C_18_H_20_N_2_O_5_
*M* *_r_* = 344.36
Triclinic, 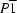

*a* = 5.5727 (1) Å
*b* = 10.8497 (1) Å
*c* = 14.2803 (2) Å
α = 100.911 (1)°
β = 96.532 (1)°
γ = 90.237 (1)°
*V* = 842.01 (2) Å^3^
*Z* = 2
Mo *K*α radiation
μ = 0.10 mm^−1^
*T* = 100 K
0.36 × 0.20 × 0.17 mm

#### Data collection

Bruker SMART APEXII CCD area-detector diffractometer
Absorption correction: multi-scan (**SADABS**; Bruker, 2005 ▶) *T*_min_ = 0.965, *T*_max_ = 0.983
21972 measured reflections
4888 independent reflections
3893 reflections with *I* > 2σ(*I*)
*R*_int_ = 0.038

#### Refinement

*R*[*F* ^2^ > 2σ(*F* ^2^)] = 0.041
*wR*(*F* ^2^) = 0.103
*S* = 1.03
4888 reflections
229 parameters
H-atom parameters constrained
Δρ_max_ = 0.40 e Å^−3^
Δρ_min_ = −0.23 e Å^−3^

### 

Data collection: *APEX2* (Bruker, 2005 ▶); cell refinement: *SAINT* (Bruker, 2005 ▶); data reduction: *SAINT*; program(s) used to solve structure: *SHELXTL* (Sheldrick, 2008 ▶); program(s) used to refine structure: *SHELXTL*; molecular graphics: *SHELXTL*; software used to prepare material for publication: *SHELXTL* and *PLATON* (Spek, 2009 ▶).

## Supplementary Material

Crystal structure: contains datablocks global, I. DOI: 10.1107/S1600536809042433/ci2940sup1.cif

Structure factors: contains datablocks I. DOI: 10.1107/S1600536809042433/ci2940Isup2.hkl

Additional supplementary materials:  crystallographic information; 3D view; checkCIF report

## Figures and Tables

**Table 1:** Hydrogen-bond geometry (Å, °)

*D*—H⋯*A*	*D*—H	H⋯*A*	*D*⋯*A*	*D*—H⋯*A*
C16—H16*A*⋯O1^i^	0.96	2.57	3.2408 (16)	127
C16—H16*A*⋯*Cg*1^i^	0.96	2.91	3.7511 (14)	147

Symmetry code: (i) 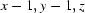. *Cg*1 is the centroid of the C1–C6 phenyl ring.

**Fig. 1.**
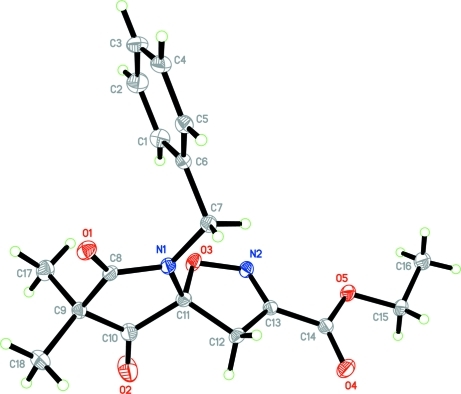
The molecular structure of the title compound, showing 50% probability displacement ellipsoids for non-H atoms and the atom-numbering scheme.

**Fig. 2.**
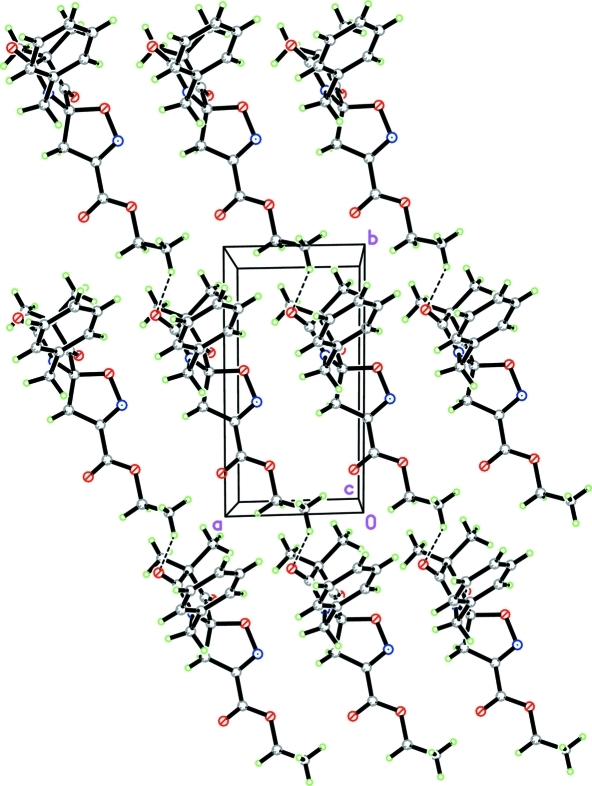
The crystal structure of the title compound, viewed along the c axis, showing chains along the [110]. Hydrogen bonds are shown as dashed lines.

## supplementary crystallographic information

### Comment

Dysidamide is a novel metabolite from a red sea sponge *Lamellodysidea herbacea* (Carmely *et al.*, 1990; Manero *et al.*, 2006; Sauleau & Bourguet-Kondracki, 2005). This hexachloro pyrrolidinone metabolite displayed remarkable biological activities such as cytotoxic activity for mesencephalic and cortical murine neuronal cell culture. We have synthesized the title compound with a spiro structure in the ring which is rare in nature, which eventually can be used as a multi-step syntheses of this marine metabolite, dysidamide, and its structure is reported here.

In the title compound (Fig. 1), the pyrrolidine ring (N1/C8–C11) adopts an envelope conformation (Boeyens, 1978; Cremer & Pople, 1975) with puckering parameters of Q = 0.1375 (12) Å and φ = 80.1 (5)°. Atom C9 deviates from the least-square plane through the remaining four atoms by 0.222 (2) Å. In contrast, the pyrrolidine ring is approximately planar in the molecular structure of 3,3-dimethylpyrrolidine-2,4-dione (Hamzah *et al.*, 2006) due to the absence of bulky groups. The dihydroisoxazole ring (C11—C13/N2/O3) is essentially planar, with a maximum deviation of 0.041 (1) Å for atom C11, and a N2–O3–C11–C12 torsion angle of -6.70 (11)°. The benzene ring (C1–C6) forms a dihedral angle of 65.19 (6)° with the dihydroisoxazole ring. Bond lengths (Allen *et al.*, 1987) and angles are within normal ranges.

In the crystal structure (Fig. 2), neighbouring molecules are linked into one-dimensional chains along the [1 1 0] by intermolecular C16—H16A···O1 hydrogen bonds and weak C16—H16A···*Cg*1 interactions (Table 1).

### Experimental

Hydroximoyl chloride (800 mg, 5.28 mmol) was dissolved in diethyl ether (100 ml) at 273 K. *N*-protected-5-methylene-pyrrolidine-2,4-dione (1.00 g, 4.36 mmol) was then added. To this mixture 5.28 ml (0.5 *M*, 10.56 mmol) of triethylamine solution in ether was added dropwise at a rate of 8 to 10 drops/min over 4 h, then kept stirring overnight. The mixture was then quenched by addition of HCl (100 ml, 2.0 N) and partitioned against ether (4 *x* 60 ml). The combined organic phases were washed with NaHCO_3_ (100 ml) and water (2 *x* 100 ml), then dried with MgSO_4_, and concentrated *in vacuo* (15 mbar) to give a yellowish oil, which was chromatographed to give 960 mg of colourless solid. Crystallization from diethyl ether gave analytically and spectroscopically pure spiroisoxazoline (860 mg, 57%) as colourless crystals (m.p. 372–373 K).

### Refinement

H atoms were placed in calculated positions, with C—H = 0.93–0.97 Å, and refined using a riding model, with *U*_iso_ = 1.2 or 1.5 *U*_eq_(C). A rotating group model was used for the methyl groups.

### Crystal data

**Table:**

C_18_H_20_N_2_O_5_	*Z* = 2
*M**_r_* = 344.36	*F*(000) = 364
Triclinic, *P*1	*D*_x_ = 1.358 Mg m^−^^3^
Hall symbol: -P 1	Mo *K*α radiation, λ = 0.71073 Å
*a* = 5.5727 (1) Å	Cell parameters from 6232 reflections
*b* = 10.8497 (1) Å	θ = 2.2–31.8°
*c* = 14.2803 (2) Å	µ = 0.10 mm^−^^1^
α = 100.911 (1)°	*T* = 100 K
β = 96.532 (1)°	Plate, colourless
γ = 90.237 (1)°	0.36 × 0.20 × 0.17 mm
*V* = 842.01 (2) Å^3^	

### Data collection

**Table:**

Bruker SMART APEXII CCD area-detector diffractometer	4888 independent reflections
Radiation source: fine-focus sealed tube	3893 reflections with *I* > 2σ(*I*)
graphite	*R*_int_ = 0.038
φ and ω scans	θ_max_ = 30.0°, θ_min_ = 1.9°
Absorption correction: multi-scan (*SADABS*; Bruker, 2005)	*h* = −7→7
*T*_min_ = 0.965, *T*_max_ = 0.983	*k* = −15→15
21972 measured reflections	*l* = −20→20

### Refinement

**Table:**

Refinement on *F*^2^	Primary atom site location: structure-invariant direct methods
Least-squares matrix: full	Secondary atom site location: difference Fourier map
*R*[*F*^2^ > 2σ(*F*^2^)] = 0.041	Hydrogen site location: inferred from neighbouring sites
*wR*(*F*^2^) = 0.103	H-atom parameters constrained
*S* = 1.03	*w* = 1/[σ^2^(*F*_o_^2^) + (0.0442*P*)^2^ + 0.2829*P*] where *P* = (*F*_o_^2^ + 2*F*_c_^2^)/3
4888 reflections	(Δ/σ)_max_ = 0.001
229 parameters	Δρ_max_ = 0.40 e Å^−^^3^
0 restraints	Δρ_min_ = −0.23 e Å^−^^3^

### Special details

**Table:**

Experimental. The crystal was placed in the cold stream of an Oxford Cyrosystems Cobra open-flow nitrogen cryostat (Cosier & Glazer, 1986) operating at 100.0 (1) K.
Geometry. All e.s.d.'s (except the e.s.d. in the dihedral angle between two l.s. planes) are estimated using the full covariance matrix. The cell e.s.d.'s are taken into account individually in the estimation of e.s.d.'s in distances, angles and torsion angles; correlations between e.s.d.'s in cell parameters are only used when they are defined by crystal symmetry. An approximate (isotropic) treatment of cell e.s.d.'s is used for estimating e.s.d.'s involving l.s. planes.
Refinement. Refinement of *F*^2^ against ALL reflections. The weighted *R*-factor *wR* and goodness of fit *S* are based on *F*^2^, conventional *R*-factors *R* are based on *F*, with *F* set to zero for negative *F*^2^. The threshold expression of *F*^2^ > σ(*F*^2^) is used only for calculating *R*-factors(gt) *etc*. and is not relevant to the choice of reflections for refinement. *R*-factors based on *F*^2^ are statistically about twice as large as those based on *F*, and *R*- factors based on ALL data will be even larger.

### Fractional atomic coordinates and isotropic or equivalent isotropic displacement parameters (Å^2^)

**Table:**

	*x*	*y*	*z*	*U*_iso_*/*U*_eq_	
O1	0.53030 (15)	0.76962 (8)	0.26077 (6)	0.01793 (18)	
O2	0.1624 (2)	0.63031 (10)	0.50066 (7)	0.0355 (3)	
O3	−0.11125 (14)	0.55167 (8)	0.30096 (6)	0.01750 (18)	
O4	0.06035 (17)	0.15205 (8)	0.32887 (7)	0.0278 (2)	
O5	−0.30443 (15)	0.17804 (8)	0.25016 (6)	0.01902 (18)	
N1	0.27928 (17)	0.60309 (9)	0.26453 (7)	0.01435 (19)	
N2	−0.20831 (17)	0.42836 (9)	0.28294 (7)	0.0160 (2)	
C1	−0.0217 (2)	0.69660 (12)	0.10602 (9)	0.0202 (2)	
H1A	−0.1142	0.6866	0.1545	0.024*	
C2	−0.0965 (2)	0.77651 (13)	0.04324 (10)	0.0243 (3)	
H2A	−0.2382	0.8205	0.0505	0.029*	
C3	0.0384 (2)	0.79100 (13)	−0.03008 (9)	0.0243 (3)	
H3A	−0.0132	0.8441	−0.0720	0.029*	
C4	0.2504 (2)	0.72606 (12)	−0.04063 (9)	0.0214 (2)	
H4A	0.3410	0.7349	−0.0899	0.026*	
C5	0.3269 (2)	0.64765 (11)	0.02275 (8)	0.0172 (2)	
H5A	0.4704	0.6052	0.0161	0.021*	
C6	0.1919 (2)	0.63167 (11)	0.09602 (8)	0.0155 (2)	
C7	0.2811 (2)	0.54453 (11)	0.16325 (8)	0.0170 (2)	
H7A	0.1793	0.4690	0.1490	0.020*	
H7B	0.4443	0.5202	0.1522	0.020*	
C8	0.39757 (19)	0.71552 (10)	0.30263 (8)	0.0135 (2)	
C9	0.3302 (2)	0.76286 (10)	0.40337 (8)	0.0145 (2)	
C10	0.2086 (2)	0.64820 (11)	0.42404 (8)	0.0181 (2)	
C11	0.1512 (2)	0.55087 (10)	0.33003 (8)	0.0141 (2)	
C12	0.2001 (2)	0.41513 (11)	0.33980 (9)	0.0160 (2)	
H12A	0.3234	0.3795	0.3007	0.019*	
H12B	0.2479	0.4085	0.4061	0.019*	
C13	−0.0410 (2)	0.35410 (10)	0.30338 (8)	0.0137 (2)	
C14	−0.0892 (2)	0.21729 (11)	0.29615 (8)	0.0161 (2)	
C15	−0.3570 (2)	0.04300 (11)	0.23740 (9)	0.0218 (3)	
H15A	−0.3953	0.0220	0.2972	0.026*	
H15B	−0.2181	−0.0044	0.2177	0.026*	
C16	−0.5689 (2)	0.01234 (12)	0.16140 (10)	0.0248 (3)	
H16A	−0.6069	−0.0760	0.1504	0.037*	
H16B	−0.5296	0.0347	0.1029	0.037*	
H16C	−0.7059	0.0587	0.1822	0.037*	
C17	0.1416 (2)	0.86566 (12)	0.39864 (10)	0.0217 (2)	
H17A	0.0040	0.8322	0.3542	0.033*	
H17B	0.2116	0.9352	0.3776	0.033*	
H17C	0.0917	0.8936	0.4612	0.033*	
C18	0.5478 (2)	0.80908 (12)	0.47626 (9)	0.0214 (2)	
H18A	0.6656	0.7446	0.4745	0.032*	
H18B	0.4975	0.8291	0.5394	0.032*	
H18C	0.6177	0.8827	0.4609	0.032*	

### Atomic displacement parameters (Å^2^)

**Table:**

	*U*^11^	*U*^22^	*U*^33^	*U*^12^	*U*^13^	*U*^23^
O1	0.0182 (4)	0.0173 (4)	0.0202 (4)	−0.0018 (3)	0.0037 (3)	0.0074 (3)
O2	0.0614 (7)	0.0298 (5)	0.0160 (5)	−0.0177 (5)	0.0121 (5)	0.0021 (4)
O3	0.0153 (4)	0.0126 (4)	0.0253 (4)	−0.0012 (3)	0.0016 (3)	0.0058 (3)
O4	0.0284 (5)	0.0157 (4)	0.0369 (6)	−0.0009 (4)	−0.0106 (4)	0.0078 (4)
O5	0.0207 (4)	0.0128 (4)	0.0229 (4)	−0.0043 (3)	−0.0033 (3)	0.0053 (3)
N1	0.0194 (4)	0.0124 (4)	0.0119 (4)	−0.0020 (3)	0.0032 (3)	0.0030 (3)
N2	0.0186 (5)	0.0126 (5)	0.0170 (5)	−0.0030 (3)	0.0018 (4)	0.0037 (4)
C1	0.0182 (5)	0.0228 (6)	0.0203 (6)	−0.0005 (4)	0.0030 (4)	0.0049 (5)
C2	0.0185 (6)	0.0255 (7)	0.0281 (7)	0.0033 (5)	−0.0019 (5)	0.0062 (5)
C3	0.0293 (6)	0.0235 (6)	0.0197 (6)	−0.0010 (5)	−0.0063 (5)	0.0089 (5)
C4	0.0284 (6)	0.0218 (6)	0.0145 (5)	−0.0033 (5)	0.0013 (5)	0.0051 (5)
C5	0.0197 (5)	0.0166 (5)	0.0148 (5)	0.0004 (4)	0.0025 (4)	0.0018 (4)
C6	0.0190 (5)	0.0140 (5)	0.0128 (5)	−0.0022 (4)	−0.0001 (4)	0.0019 (4)
C7	0.0238 (6)	0.0148 (5)	0.0128 (5)	0.0005 (4)	0.0039 (4)	0.0024 (4)
C8	0.0141 (5)	0.0118 (5)	0.0151 (5)	0.0011 (4)	0.0004 (4)	0.0043 (4)
C9	0.0159 (5)	0.0134 (5)	0.0140 (5)	−0.0021 (4)	0.0016 (4)	0.0022 (4)
C10	0.0218 (5)	0.0166 (5)	0.0159 (5)	−0.0020 (4)	0.0037 (4)	0.0026 (4)
C11	0.0153 (5)	0.0140 (5)	0.0138 (5)	−0.0016 (4)	0.0014 (4)	0.0048 (4)
C12	0.0168 (5)	0.0135 (5)	0.0186 (5)	−0.0009 (4)	−0.0001 (4)	0.0066 (4)
C13	0.0165 (5)	0.0135 (5)	0.0117 (5)	−0.0013 (4)	0.0024 (4)	0.0032 (4)
C14	0.0193 (5)	0.0150 (5)	0.0142 (5)	−0.0019 (4)	0.0013 (4)	0.0034 (4)
C15	0.0288 (6)	0.0115 (5)	0.0242 (6)	−0.0051 (5)	−0.0043 (5)	0.0053 (5)
C16	0.0271 (6)	0.0180 (6)	0.0269 (7)	−0.0045 (5)	−0.0058 (5)	0.0041 (5)
C17	0.0201 (6)	0.0181 (6)	0.0261 (6)	0.0038 (4)	0.0036 (5)	0.0019 (5)
C18	0.0198 (5)	0.0246 (6)	0.0179 (6)	−0.0032 (5)	−0.0007 (4)	0.0014 (5)

### Geometric parameters (Å, °)

**Table:**

O1—C8	1.2160 (13)	C7—H7B	0.97
O2—C10	1.2022 (15)	C8—C9	1.5228 (16)
O3—N2	1.4074 (12)	C9—C10	1.5072 (16)
O3—C11	1.4748 (13)	C9—C18	1.5218 (16)
O4—C14	1.2065 (14)	C9—C17	1.5413 (16)
O5—C14	1.3273 (14)	C10—C11	1.5436 (16)
O5—C15	1.4659 (14)	C11—C12	1.5281 (16)
N1—C8	1.3708 (14)	C12—C13	1.4891 (16)
N1—C11	1.4333 (14)	C12—H12A	0.97
N1—C7	1.4666 (14)	C12—H12B	0.97
N2—C13	1.2793 (15)	C13—C14	1.4894 (16)
C1—C2	1.3933 (18)	C15—C16	1.5006 (17)
C1—C6	1.3935 (16)	C15—H15A	0.97
C1—H1A	0.93	C15—H15B	0.97
C2—C3	1.3882 (19)	C16—H16A	0.96
C2—H2A	0.93	C16—H16B	0.96
C3—C4	1.3861 (19)	C16—H16C	0.96
C3—H3A	0.93	C17—H17A	0.96
C4—C5	1.3897 (17)	C17—H17B	0.96
C4—H4A	0.93	C17—H17C	0.96
C5—C6	1.3918 (16)	C18—H18A	0.96
C5—H5A	0.93	C18—H18B	0.96
C6—C7	1.5170 (16)	C18—H18C	0.96
C7—H7A	0.97		
			
N2—O3—C11	109.86 (8)	N1—C11—O3	110.01 (9)
C14—O5—C15	115.70 (9)	N1—C11—C12	117.91 (9)
C8—N1—C11	115.03 (9)	O3—C11—C12	104.33 (9)
C8—N1—C7	121.36 (9)	N1—C11—C10	102.13 (9)
C11—N1—C7	123.59 (9)	O3—C11—C10	107.65 (9)
C13—N2—O3	108.85 (9)	C12—C11—C10	114.56 (9)
C2—C1—C6	119.84 (11)	C13—C12—C11	101.13 (9)
C2—C1—H1A	120.1	C13—C12—H12A	111.5
C6—C1—H1A	120.1	C11—C12—H12A	111.5
C3—C2—C1	120.60 (12)	C13—C12—H12B	111.5
C3—C2—H2A	119.7	C11—C12—H12B	111.5
C1—C2—H2A	119.7	H12A—C12—H12B	109.4
C4—C3—C2	119.76 (11)	N2—C13—C12	115.35 (10)
C4—C3—H3A	120.1	N2—C13—C14	121.88 (10)
C2—C3—H3A	120.1	C12—C13—C14	122.68 (10)
C3—C4—C5	119.69 (11)	O4—C14—O5	125.57 (11)
C3—C4—H4A	120.2	O4—C14—C13	120.96 (10)
C5—C4—H4A	120.2	O5—C14—C13	113.47 (10)
C4—C5—C6	121.01 (11)	O5—C15—C16	107.20 (10)
C4—C5—H5A	119.5	O5—C15—H15A	110.3
C6—C5—H5A	119.5	C16—C15—H15A	110.3
C5—C6—C1	119.10 (11)	O5—C15—H15B	110.3
C5—C6—C7	119.32 (10)	C16—C15—H15B	110.3
C1—C6—C7	121.58 (10)	H15A—C15—H15B	108.5
N1—C7—C6	112.30 (9)	C15—C16—H16A	109.5
N1—C7—H7A	109.1	C15—C16—H16B	109.5
C6—C7—H7A	109.1	H16A—C16—H16B	109.5
N1—C7—H7B	109.1	C15—C16—H16C	109.5
C6—C7—H7B	109.1	H16A—C16—H16C	109.5
H7A—C7—H7B	107.9	H16B—C16—H16C	109.5
O1—C8—N1	124.73 (10)	C9—C17—H17A	109.5
O1—C8—C9	125.87 (10)	C9—C17—H17B	109.5
N1—C8—C9	109.37 (9)	H17A—C17—H17B	109.5
C10—C9—C18	112.41 (10)	C9—C17—H17C	109.5
C10—C9—C8	101.90 (9)	H17A—C17—H17C	109.5
C18—C9—C8	113.02 (9)	H17B—C17—H17C	109.5
C10—C9—C17	108.72 (9)	C9—C18—H18A	109.5
C18—C9—C17	111.74 (10)	C9—C18—H18B	109.5
C8—C9—C17	108.54 (9)	H18A—C18—H18B	109.5
O2—C10—C9	127.32 (11)	C9—C18—H18C	109.5
O2—C10—C11	123.09 (11)	H18A—C18—H18C	109.5
C9—C10—C11	109.58 (9)	H18B—C18—H18C	109.5
			
C11—O3—N2—C13	4.15 (12)	C8—N1—C11—O3	112.65 (10)
C6—C1—C2—C3	0.8 (2)	C7—N1—C11—O3	−65.65 (13)
C1—C2—C3—C4	−0.4 (2)	C8—N1—C11—C12	−127.96 (11)
C2—C3—C4—C5	−0.45 (19)	C7—N1—C11—C12	53.74 (15)
C3—C4—C5—C6	0.98 (18)	C8—N1—C11—C10	−1.45 (12)
C4—C5—C6—C1	−0.62 (18)	C7—N1—C11—C10	−179.76 (10)
C4—C5—C6—C7	179.60 (11)	N2—O3—C11—N1	120.68 (9)
C2—C1—C6—C5	−0.26 (18)	N2—O3—C11—C12	−6.70 (11)
C2—C1—C6—C7	179.51 (11)	N2—O3—C11—C10	−128.80 (9)
C8—N1—C7—C6	−55.05 (14)	O2—C10—C11—N1	−169.17 (13)
C11—N1—C7—C6	123.15 (11)	C9—C10—C11—N1	10.03 (12)
C5—C6—C7—N1	130.98 (11)	O2—C10—C11—O3	75.00 (15)
C1—C6—C7—N1	−48.79 (15)	C9—C10—C11—O3	−105.80 (10)
C11—N1—C8—O1	174.18 (10)	O2—C10—C11—C12	−40.52 (17)
C7—N1—C8—O1	−7.48 (17)	C9—C10—C11—C12	138.68 (10)
C11—N1—C8—C9	−7.56 (13)	N1—C11—C12—C13	−116.02 (10)
C7—N1—C8—C9	170.79 (9)	O3—C11—C12—C13	6.31 (11)
O1—C8—C9—C10	−168.78 (11)	C10—C11—C12—C13	123.75 (10)
N1—C8—C9—C10	12.98 (12)	O3—N2—C13—C12	0.42 (13)
O1—C8—C9—C18	−47.93 (15)	O3—N2—C13—C14	177.19 (9)
N1—C8—C9—C18	133.83 (10)	C11—C12—C13—N2	−4.48 (13)
O1—C8—C9—C17	76.60 (14)	C11—C12—C13—C14	178.79 (10)
N1—C8—C9—C17	−101.64 (10)	C15—O5—C14—O4	−1.42 (17)
C18—C9—C10—O2	44.01 (18)	C15—O5—C14—C13	177.48 (9)
C8—C9—C10—O2	165.29 (13)	N2—C13—C14—O4	−168.93 (12)
C17—C9—C10—O2	−80.23 (16)	C12—C13—C14—O4	7.60 (17)
C18—C9—C10—C11	−135.14 (10)	N2—C13—C14—O5	12.11 (16)
C8—C9—C10—C11	−13.86 (12)	C12—C13—C14—O5	−171.37 (10)
C17—C9—C10—C11	100.62 (11)	C14—O5—C15—C16	−163.82 (10)

### Hydrogen-bond geometry (Å, °)

**Table:**

*D*—H···*A*	*D*—H	H···*A*	*D*···*A*	*D*—H···*A*
C16—H16A···O1^i^	0.96	2.57	3.2408 (16)	127
C16—H16A···Cg1^i^	0.96	2.91	3.7511 (14)	147

Symmetry codes: (i) *x*−1, *y*−1, *z*.
